# Supplementary material for: Iterative improvement in the automatic modular design of robot swarms
Source: PeerJ Comput Sci. 2020 Dec 7;6:e322. doi: 10.7717/peerj-cs.322 (PMC7924708; doi:10.7717/peerj-cs.322)
Supplement: Supplemental Information 3 [file peerj-cs-06-322-s003.zip › argos3/doc/api/standalone/a00504_source.html]

ARGoS: plugins/robots/generic/control\_interface/ci\_pan\_tilt\_camera\_actuator.cpp Source File


- Main Page
- Related Pages
- Namespaces
- Classes
- Files

- File List
- File Members

# plugins/robots/generic/control\_interface/ci\_pan\_tilt\_camera\_actuator.cpp

Go to the documentation of this file.

```
00001 
00007 #include "ci_pan_tilt_camera_actuator.h"
00008 
00009 #ifdef ARGOS_WITH_LUA
00010 #include <argos3/core/wrappers/lua/lua_utility.h>
00011 #endif
00012 
00013 namespace argos {
00014 
00015    /****************************************/
00016    /****************************************/
00017 
00018 #ifdef ARGOS_WITH_LUA
00019    /*
00020     * The stack must have one value:
00021     * 1. the pan (in radians)
00022     */
00023    int LuaSetPan(lua_State* pt_lua_state) {
00024       /* Check parameters */
00025       if(lua_gettop(pt_lua_state) != 1) {
00026          return luaL_error(pt_lua_state, "robot.pan_tilt_camera.set_pan() expects 1 argument");
00027       }
00028       luaL_checktype(pt_lua_state, 1, LUA_TNUMBER);
00029       /* Perform action */
00030       CLuaUtility::GetDeviceInstance<CCI_PanTiltCameraActuator>(pt_lua_state, "pan_tilt_camera")->
00031          SetPan(CRadians(lua_tonumber(pt_lua_state, 1)));
00032       return 0;
00033    }
00034 #endif
00035    
00036    /****************************************/
00037    /****************************************/
00038 
00039 #ifdef ARGOS_WITH_LUA
00040    /*
00041     * The stack must have one value:
00042     * 1. the tilt (in radians)
00043     */
00044    int LuaSetTilt(lua_State* pt_lua_state) {
00045       /* Check parameters */
00046       if(lua_gettop(pt_lua_state) != 1) {
00047          return luaL_error(pt_lua_state, "robot.pan_tilt_camera.set_tilt() expects 1 argument");
00048       }
00049       luaL_checktype(pt_lua_state, 1, LUA_TNUMBER);
00050       /* Perform action */
00051       CLuaUtility::GetDeviceInstance<CCI_PanTiltCameraActuator>(pt_lua_state, "pan_tilt_camera")->
00052          SetTilt(CRadians(lua_tonumber(pt_lua_state, 1)));
00053       return 0;
00054    }
00055 #endif
00056    
00057    /****************************************/
00058    /****************************************/
00059 
00060 #ifdef ARGOS_WITH_LUA
00061    void CCI_PanTiltCameraActuator::CreateLuaState(lua_State* pt_lua_state) {
00062       CLuaUtility::OpenRobotStateTable(pt_lua_state, "pan_tilt_camera");
00063       CLuaUtility::AddToTable(pt_lua_state, "_instance", this);
00064       CLuaUtility::AddToTable(pt_lua_state, "set_pan", &LuaSetPan);
00065       CLuaUtility::AddToTable(pt_lua_state, "set_tilt", &LuaSetTilt);
00066       CLuaUtility::CloseRobotStateTable(pt_lua_state);
00067    }
00068 #endif
00069 
00070    /****************************************/
00071    /****************************************/
00072 
00073 }
```

---

Generated on 10 Jul 2018 for ARGoS by 
 1.6.1
